# Determinants of Salt-Restriction-Spoon Using Behavior in China: Application of the Health Belief Model

**DOI:** 10.1371/journal.pone.0083262

**Published:** 2013-12-20

**Authors:** Juan Chen, Yixing Liao, Zhuoting Li, Ye Tian, Shuaishuai Yang, Chao He, Dahong Tu, Xinying Sun

**Affiliations:** 1 Department of Social Medicine and Health Education, School of Public Health, Peking University, Beijing, China; 2 VIP Medical Service Department, Peking Union Medical College Hospital, Beijing, China; 3 China Center for Health Development Studies, School of Public Health, Peking University, Beijing, China; 4 Department of Health Education, Shun Yi Center for Disease Prevention and Control, Beijing, China; 5 Community Medical Center, Beijing Shijitan Hospital, Beijing, China; The Ohio State University, United States of America

## Abstract

**Background:**

The two-gram salt-restriction-spoons, which can be used to reduce the salt intake of people, had been handed out for free by the Chinese government to the citizens several years ago, but only a small fraction of residents use such a spoon currently. Since no studies have been conducted to investigate relevant influencing factors, this study was designed to explore the determinants of salt-restriction-spoon using behavior (SRB) in China.

**Methods:**

This cross-sectional study was conducted in Beijing, China. Altogether 269 rural residents and 244 urban residents aged over 18 were selected by convenience sampling method in 2012. Variables measured in a questionnaire designed according to the Health Belief Model (HBM) included socio-demographics, perceived susceptibility, perceived severity, perceived benefits, perceived objective barriers, perceived subjective barriers, self-efficacy, knowledge of hypertension, cues to action, and SRB. Answers to the questionnaire were obtained from all the participants, and 24-hour urine samples were collected to determine the 24-hour urinary sodium excretion (24HUNa). Path analyses were used to explore the determinants of SRB.

**Results:**

Approximately 22.7% and 45.3% of residents used a salt-restriction-spoon everyday in the rural and urban areas, respectively. The average 24HUNa was 211.19±98.39 mmol for rural residents and 109.22±58.18 mmol for urban residents. Path analyses shown that perceived objective barriers, perceived benefits, perceived severity, knowledge and age were related to SRB and 24HUNa for both rural and urban participants, among which perceived objective barrier (β =  − 0.442 and β =  − 0.543, respectively) was the most important determinant.

**Conclusion:**

Improvement of the current salt-restriction-spoon and education on the right usage of the salt-restriction-spoon, the severity of hypertension, and the benefit of salt reduction are necessary, especially among those who are relatively young but at risk of hypertension, those who have lower education levels, and those who live in the rural areas.

## Introduction

Hypertension has affected a large number of people all over the world. The overall prevalence of hypertension among adults aged over 18 was 33.5% in China in 2010 [1]. According to the results of many other studies, hypertension is a major risk factor for stroke [2], renal disease [3] and heart failure [4].

There are several factors that contribute to the development of hypertension, such as old age, obesity, sedentary lifestyle, salt intake, lack of physical activity, imbalanced diet, some socioeconomic factors and psychological factors, among which, salt intake is an relatively major one [5]. A number of studies have demonstrated that a reduction in salt intake can lower blood pressure significantly [6], [7], and help to enhance the curative effect of antihypertensive drugs and non-pharmacological treatments for high blood pressure [8], [9]. Salt reduction has been emphasized by the World Health Organization (WHO) and many countries’ prevention and cure guideline of hypertension as a critical part of the prevention and treatment of hypertension [10], [11], [12].

The Chinese National Survey conducted in 2002 shown that the average daily salt consumption of Chinese people was 12 grams among Chinese residents, and 81.6% of residents (83.8% of rural residents, and 76.8% of urban residents) consumed more than the recommended amount of 6g [13]. Previous study has shown that most dietary sodium of Chinese people was from the salt added in home cooking, which differed largely from the situation in western countries, whose source of sodium was mainly from eating out or eating prepared foods, therefore the effort of salt reduction in China should be focus on home cooking [14].

As a measure to reduce salt, many local governments in China have handed out two-gram salt-restriction-spoons among citizens, and most people in Beijing had received such spoons by 2008. Those who have received such a spoon were supposed to have been educated to eat less than 6 g salt per day. With a 5 cm handle, and a 1.1 cm caliber, this spoon was designed to hold 2g kitchen salt and thus 3 spoons of salt equals to 6 g salt. The reason for the wide application of such a spoon in China is that it can help people calculate the amount of salt in home cooking. For example, a family with three members should eat no more than 18 g salt per day. If this family does not eat salt at breakfast, they can eat 9 g salt at lunch or supper, that is, the housewife can use 4 to 5 spoons of salt per meal.

It has been proved that enhancing the correctly using rate of salt-restriction-spoon can reduce the salt intake and blood pressure of people. For example, the studies of Li Yuqing [15] and Yang Jun [16] found that using a salt-restriction-spoon correctly or eating low sodium salt can reduce the amount of salt intake and lower the level of blood pressure; the effect would be more obvious if the two actions were taken simultaneously. However, many people who have received a salt-restriction-spoon do not use it or cannot use it correctly. Survey conducted in 2011 has shown that the owning rate and using rate of salt-restriction-spoon among those who received it in 2007−2008 in Beijing were only 61.8% and 47.7%, respectively, and only 17.1% could use it correctly (use it according to the standard of 6 g/day/person) [17]. Our research group found in 2011 that the receiving rate and using rate of salt-restriction-spoon in Beijing were 72.1% and 32.0%, respectively [18].

It’s not clear why people are not willing to use this spoon. A better understanding of important psychosocial variables that influence salt-restriction-spoon using behavior (SRB) is in need so as to design more effective intervention strategies. So far, there is no previous systematic exploration of the determinants of residents’ SRB, with the exception of some sporadic studies on salt-restriction behavior carried among hypertensive patients [19], [20], [21], [22]. Applications of health behavior models in SRB prediction have been largely lacking. The Health Belief Model (HBM) was selected as the theoretical framework based on its ability to successfully predict health behaviors [23], such as functional bread consumption behavior [24] and iron-fortified soy sauce consumption behavior [25].

According to the HBM [26], individuals will engage in positive health behaviors when they see themselves as susceptible to illness (perceived susceptibility), perceive the severity of the consequences of such an illness (perceived severity), see the benefits of acting healthily (perceived benefits), and believe that the behavior’s benefits exceed the costs of undertaking it (perceived barriers). Cues to action are considered modifying variables that influence behavior by prompting actions, and self-efficacy refers to the confidence in one’s ability to successfully perform the action.

We anticipate that individuals who are older, with better knowledge of hypertension, a higher education level, higher income and those who get access to more cues to action would perceive more susceptibility to hypertension and more severity of hypertension, and understand the benefits of salt-restriction better. These factors, in turn, may reduce the barriers faced by these individuals and enhance their confidence in salt-restriction, which may lead to better SRB and lower 24-hour urine sodium (24HUNa).

## Methods

### Ethics statement

Before conducting the survey, written informed consent was obtained from each participant in accordance with the ethical standards of the Helsinki Declaration. The study has received approval from the Peking University Institutional Review Board and the approval number is IRB00001052-12010.

### Sample

This is a cross-sectional survey carried out in two communities of an urban district and in two villages in Beijing. With high density of population and dominated with nonfarm economy, the two communities were hereinafter referred to as the urban area, and people living in these two communities were hereinafter referred to as urban residents. Likewise, with low density of population and dominated with farm economy, the two villages were hereinafter referred to as the rural area, and people living in these two villages were hereinafter referred to as rural residents [27]. Convenience sampling method was used to select two districts in Beijing, and then two communities/villages in each district. Finally, based on the principle of voluntary participation, convenience sampling method was used to enroll 200 residents who were solely or jointly responsible for their home cooking from each community/village to finish a questionnaire survey, thus a total of 800 residents were enrolled in this study. Among the 800 residents, 240 who did not collect urine samples and 48 who collected less than 60% of the 24-hour urine samples were excluded, leaving 512 who finished the questionnaire as well as collected no less than 60% of the 24-hour urine being included in the data analysis.

With the calculation formula 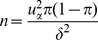, in which *π* referred to the using frequency of salt-restriction-spoon, namely 32% [18], *δ* = 0.05, *u_0.05_*  = 1.96, the sample size was calculated to be *n* = 334. Given that the whole study was a community intervention trial with an intervention group and a control group, the sample size was doubled to be *2n* = 668, and was expanded to be 800 for the consideration of invalid cases or other reasons. However, the analysis in this manuscript only involved with the cross-sectional part of this study, therefore, a sample size of 512 is enough. For constructing a path model, Klein recommended a minimum of 10 cases for every parameter estimated and only paths with significance in univariate analysis need to be connected [28]. Therefore, less than 20 parameters were included in the analysis, and a sample size of 200 would be enough for each model.

### Questionnaire

A self-designed questionnaire was structured based on the HBM, in which participants’ SRB, demographic characteristics (age, degree of education, family income, and place of residence), perceived susceptibility to hypertension, perceived severity of hypertension, perceived benefit of salt reduction, perceived objective barriers in using salt-restriction spoon (the barriers related to the design defect and the usage of the salt-restriction-spoon, in which the design defect means the features that make the spoons inconvenient for use, for example, short handle, small caliber, etc.), perceived subjective barriers in using salt-restriction spoon (the barriers related to the negative feeling of salt reduction), self-efficacy, knowledge of hypertension and cues to action (the cues that may prompt someone to use a salt-restriction-spoon, for example, a woman with a hypertensive father may be more likely to use a salt-restriction-spoon) were tested. This instrument has good reliability and validity (see Table 1).

**Table 1:** Reliability and Construct Validity of the questionnaire.

Variables	Items	Range	Item example	Cronbach α	% of variance*
Salt-restriction-spoon using behavior (SRB)	1	1−5	How often did you use the two-gram salt-restriction-spoon (SRS) last month? (Never = 1, seldom = 2, sometimes = 3, often = 4, everyday = 5)		
Perceived susceptibility to hypertension	6	5−26	I feel my chance of getting hypertension in the future is high. (Strongly disagree = 1, disagree = 2, neutral = 3, agree = 4, strongly agree = 5) I have a history of hypertension. (No = 0, yes = 1)	0.894	9.979
Perceived severity of hypertension	6	6−30	If I suffer from hypertension, I will feel dizzy. (Strongly disagree = 1, disagree = 2, neutral = 3, agree = 4, strongly agree = 5)	0.807	4.132
Perceived benefits of salt-restriction	6	6−30	I feel that salt restriction can prevent hypertension. (Strongly disagree = 1, disagree = 2, neutral = 3, agree = 4, strongly agree = 5)	0.803	6.567
Perceived subjective barriers of salt-restriction	5	5−25	I feel that I cannot get used to less salty food. (Strongly disagree = 1, disagree = 2, neutral = 3, agree = 4, strongly agree = 5)	0.680	4.549
Perceived objective barriers of salt-restriction	9	9−45	I do not know how to calculate the correct amount of daily salt intake. (Strongly disagree = 1, disagree = 2, neutral = 3, agree = 4, strongly agree = 5)	0.838	9.874
Self-efficacy	9	9−45	I am confident in using the salt-restriction-spoon to reduce salt. (Strongly disagree = 1, disagree = 2, neutral = 3, agree = 4, strongly agree = 5)	0.912	15.752
Knowledge of hypertension	6	0−6	What is the recommended amount of daily salt intake per person? (Incorrect answer = 0, correct answer = 1)	0.766	7.507
Cues to action	5	0−5	I have been suggested to reduce salt intake by a doctor. (No = 0, yes = 1)	0.642	4.473

Note: Initial eigenvalue >1 was the criterion used for the factor analysis.

* Eight factors explain 62.833% of variance of the questionnaire cumulatively.

The questionnaire survey was finished at the participants’ home. The answers to the questionnaire were asked from the participants and filled on the questionnaires by strictly trained investigators. In order to acquire the real thoughts of the participants, the investigators were trained to avoid inducing the participants’ thoughts and to inform the participants the importance of the questionnaire survey. All the questionnaires collected by the investigators were rechecked by a quality controller.

### Urine sample collection and sodium excretion determination

Not any advice was given to the participants before collecting urine. However, patients with kidney disease were not included in this study. The 24-hour urine was collected into clean plastic containers at home from 07:00 to 07:00 in the next morning. The percentage of collected 24-hour urine was ascertained from the subjects when they returned the containers and the actual urinary volume (mL) was measured, which can be used to calculate the total 24-hour urinary volume (mL). An AC9000 electrolytic analyzer produced by Jiangsu Audicom Medical Technology and the iron selective electrode method were used to determine urinary sodium concentrations (mol/L). The 24HUNa was calculated with this formula: 24HUNa  =  Sodium concentration (mol/L) × total 24-hour urinary volume (L).

### Analysis

Data was processed by EpiData 3.0 and analyzed by SPSS 17.0. Scale and factor analyses were conducted to verify the reliability and validity of the questionnaire. Descriptive analyses were implemented to describe the demographic features, SRB, and 24HUNa. Correlation analysis was used to explore the association between variables. Path analysis was implemented by LISREL 8.70 to test the goodness of fit of the model and the association between variables in the HBM. Generally, *P* = 0.05 was set as the level of significance.

## Results

### Participant Characteristics

Table 2 shows the demographic features of all the participants. Among the 512 participants included in the analysis, 52.5% and 47.5% came from the rural and urban, respectively. The majority of rural participants were females (75.1%) with an average age of 56.29 (SD = 11.18). The majority of urban participants were females (61.3%) with an average age of 63.36 (SD = 12.07). The urban participants tended to have significantly older age (*P*<0.001), higher education level (*P*<0.001) and family income (*P*<0.001) as compared to the rural participants (See Table 2). All participants ate at home for at least one meal per day. In the weekdays, the percentages of rural participants eating at home for one, two or three meals were 3.7%, 11.9%, and 84.4%, respectively; the percentages of urban participants eating at home for one, two or three meals were 1.6%, 9.5%, and 88.9%, respectively. In the weekends, the percentages of rural participants eating at home for one, two or three meals were 2.2%, 9.3%, and 88.5%, respectively; the percentages of urban participants eating at home for one, two or three meals were 2.5%, 9.5%, and 88.0%, respectively.

**Table 2:** Demographic features of participants.

		Rural			Urban		
Demographic features	Included (n = 269)	Excluded (n = 135)	*F* value a	Included (n = 243)	Excluded (n = 153)	*F* value a	*F* value b
Age (years)			34.133***			8.117*	47.730***
18−49	64 (23.8)	71 (52.6)		26 (10.7)	28 (18.3)		
50−59	93 (34.6)	33 (24.4)		57 (23.5)	45 (29.4)		
60−69	82 (30.5)	23 (17.0)		78 (32.1)	39 (25.5)		
70+	30 (11.2)	8 (5.9)		82 (33.7)	41 (26.8)		
Education			19.778***			6.550	125.940***
Primary school graduate	75 (27.9)	24 (17.8)		18 (7.4)	21 (13.7)		
Junior high school graduate	151 (56.1)	71 (52.6)		79 (32.5)	36 (23.5)		
Senior high school graduate	39 (24.5)	27 (20.0)		79 (32.5)	53 (34.6)		
More than high school	4 (1.5)	13 (9.6)		67 (27.6)	43 (28.1)		
Income			11.211*			12.419**	82.036***
<1000	78 (29.0)	19 (14.1)		8 (3.3)	5 (3.3)		
1000−2999	87 (32.3)	55 (40.7)		70 ( 28.8)	69 (45.1)		
3000−4999	76 (28.3)	43 (31.9)		87 (35.8)	48 (31.4)		
5000+	28 (10.4)	18 (13.3)		78 (32.1)	31 (20.3)		
Gender			4.356*			0.244	11.239**
Male	67 (24.9)	47 (34.8)		94 (38.7)	63 (41.2)		
Female	202 (75.1)	88 (65.2)		149 (61.3)	90 (58.8)		

Note: ^*^ *P*<0.05; ^**^ *P*<0.01; ^***^ *P*<0.001.

a *F* value for comparing the features of included population and excluded population.

b *F* value for comparing the features of included rural residents and included urban residents.

Some demographic features of those excluded from the analysis were different from those included in the analysis. For rural residents, the ones included in the analysis were older, had poorer educational background and lower income as compared to those excluded from the analysis. For urban residents, those included in the analysis were older and with lower income than those excluded from the analysis. The main reason for no or incomplete submission of 24HUNa was that the younger residents who have higher educational level and higher income must leave home for work, making it inconvenient for them to collect 24-hour urine.

### SRB

Urban residents had significantly better SRB (P<0.001) as compared to rural residents (Table 3). Among rural residents, 22.7% (61) use a salt-restriction-spoon every day, 6.7% (18), 6.7% (18), 5.2% (14) and 58.7% (158) often, occasionally, seldom, and never use a salt-restriction-spoon, respectively; only 12.3% (33) can use it correctly. Among urban residents, 45.3% (110) use a salt-restriction-spoon every day, 11.5% (28), 11.5% (28), 12.3% (30) and 19.3% (47) often, occasionally, seldom, and never use salt-restriction-spoon, respectively; 27.6% (67) can use it correctly. There were positive correlations between the using frequency (every day to never) and the using method (correct or not) both for the rural participant (Spearman correlation = 0.311, *P*<0.01) and for the urban participants (Spearman correlation = 0.518, *P*<0.01). Binary variable is not suitable for path analysis, so the using frequency instead of the using method was chosen to represent SRB.

**Table 3:** Participants’ scores on SRB, 24HUNa, and variables of Health Belief Model.

Demographic features	Susceptibility	Severity	Objective barriers	Self-efficacy	Knowledge	Cues	SRB	24HUNa
Total	15.02±5.70	23.68±3.94	25.96±7.39	36.74±5.48	3.95±1.46	3.37±1.46	2.87±1.76	162.80±96.33
Age (years)								
18−49	13.87±5.36	23.73±3.48	27.59±7.35	35.66±5.41	3.46±1.55	3.03±1.52	2.15±1.48	181.74±109.23
50−59	14.37±5.91	24.01±4.06	26.40±7.56	37.88±5.09	3.83±1.68	3.37±1.50	2.90±1.81	177.06±102.40
60−69	15.98±5.58	24.14±3.94	25.55±7.41	37.12±5.47	4.21±1.55	3.46±1.35	2.77±1.76	168.66±80.93
70+	15.45±5.68	22.54±3.93	24.66±6.96	35.54±5.72	4.12±1.84	3.50±1.49	3.55±1.66	120.08±85.06
*F* value	3.673*	4.345**	2.984*	5.541**	4.463**	2.119	11.405^***^	10.332^***^
Education								
Lower than middle school	13.86±6.01	23.26±4.72	28.20±7.31	36.62±6.08	2.81±1.86	2.96±1.36	2.29±1.64	187.09±112.47
Junior middle school	15.16±5.65	24.05±3.92	26.20±7.51	37.11±5.24	3.87±1.59	3.23±1.56	2.79±1.79	175.15±92.66
Senior middle school	15.28±5.56	23.35±3.63	24.38±6.86	36.55±5.55	4.54±1.31	3.81±1.27	3.20±1.70	143.12±95.87
Higher than middle school	15.69±5.62	23.61±3.25	24.87±7.28	35.98±5.32	4.72±1.36	3.66±1.30	3.34±1.70	123.65±64.55
*F* value	1.742	1.318	5.379**	0.864	28.385^***^	8.789^***^	6.815^***^	9.205^***^
Income								
<1000	14.00±5.91	23.60±4.67	27.15±8.00	37.22±6.70	2.87±1.79	2.87±1.48	2.60±1.76	182.60±84.40
1000−2999	15.21±5.60	23.52±3.75	25.82±7.00	36.53±5.06	3.89±1.68	3.38±1.46	2.93±1.75	177.91±105.40
3000−4999	15.11±5.89	23.77±3.72	25.83±7.70	36.90±5.16	4.14±1.53	3.36±1.47	2.87±1.79	150.80±97.03
5000+	15.45±5.38	23.84±3.92	25.42±6.95	36.41±5.51	4.62±1.32	3.76±1.33	3.00±1.74	142.79±84.11
*F* value	1.192	0.182	0.972	0.476	20.415***	6.089***	0.903	4.980**
Areas								
rural	14.51±5.94	24.06±4.29	27.65±7.63	37.52±5.58	3.15±1.70	2.88±1.45	2.29±1.70	211.19±98.39
urban	15.60±5.39	23.32±3.47	24.09±6.65	35.86±5.24	4.84±1.08	3.91±1.27	3.51±1.60	109.22±58.18
*F* value	4.667*	5.165*	31.427^***^	11.907**	175.004^***^	72.788^***^	69.010^***^	198.353^***^

Note: ^*^ *P*<0.05; ^**^ *P*<0.01; ^***^ *P*<0.001; Scores were shown as mean±standard deviation.

Abbreviations: SRB = salt-restriction-spoon using behavior; 24HUNa = 24-hour urinary sodium.

### 24HUNa

The average 24HUNa was significantly higher for rural residents than urban residents (P<0.001, Table 3). In order to investigate the source of Na, the consumptions of kitchen salt, soy sauce, monosodium glutamate, and chicken essence were tracked for one month, the result of which shown that the proportion of Na coming from kitchen salt was similar between the rural and the urban residents (68.74% and 67.24%, respectively). That is, although the absolute amount of salt intake was different for the two groups of people, the habits for use of condiments that are very high in sodium were similar between them.

In order to explore the difference of 24HUNa between those included in and those excluded from the analysis, multivariate linear regression with gender, district, educational background, knowledge on hypertension, BMI, age, using frequency of salt-restriction-spoon, and using method of salt-restriction-spoon to be independent variables was applied to predict 24HUNa. According to the generated predicted model (24HUNa  =  246.579 – 99.683*district + 3.348*BMI + 5.875*knowledge of hypertension – 0.671*age), residents living in the rural area (*β*  =  −0.477, *P* < 0.001), with higher BMI (*β*  =  0.123, *P*  =  0.001), more knowledge of hypertension (*β*  =  0.095, *P*  =  0.027), and younger age (*β*  =  −0.080, *P*  =  0.038) had higher level of 24HUNa. No significant difference existed in the predicted 24HUNa of those included in and those excluded from the analysis both for the rural area (213.20±19.98 mmol for included ones vs 217.17±18.94 mmol for excluded ones, *P* = 0.056) and the urban area (114.09±16.48 mmol vs 113.78±17.91 mmol, *P* = 0.861).

### Scores on the variables of HBM

Table 3 shows the scores of variables among participants with different ages, education levels, family income and places of residence. Benefit and perceived subjective barriers, the scores of which were not significantly different among participants with different characteristics, are not included in this table. As depicted in Table 3, participants with older age, higher education level, more family income and from urban area had more knowledge on hypertension. Participants with lower education level and from the rural area perceived more objective barriers. Participants with higher education level, more family income and from the urban area scored higher on cues to action.

### Correlation matrix of the variables

The correlation matrix for rural participants (Table 4) shows that SRB was significantly related to most variables in the model, especially education (*r* = 0.163), income (*r* = −0.205), objective barriers (*r* =  − 0.473), and self-efficacy (*r* = 0.210). 24HUNa was significantly related to knowledge (*r* =  − 0.216). The correlation matrix for urban participants (Table 4) shows that SRB was not significantly related to education and income, but to other variables, especially age (*r* = 0.318), benefit (*r* = −0.262), subjective barriers (*r* =  − 0.183), objective barriers (*r* = −0.596), and self-efficacy (*r* = 0.236). 24HUNa was not significantly related to knowledge, but to other variables, such as age (*r* =  − 0.188) and susceptibility (*r* =  − 0.215).

**Table 4:** Correlation matrix of the variables.

	Age	Knowledge	Education	Income	Cues	Susceptibility	Severity	Benefit	Subjective barriers	Objective barriers	Self-efficacy	SRB	24HUNa
Age	1	.147^*^	−.217^**^	.000	.102	−.022	−.099	−.028	−.055	−.183^**^	.052	.318^**^	−.188^**^
Knowledge	−.047	1	.112	.083	.287^**^	.209^**^	.196^**^	.132^*^	−.129^*^	−.247^**^	.070	.088	.063
Education	−.343^**^	.278^**^	1	.230^**^	−.033	.075	.008	−.008	−.059	.041	.018	−.105	.155^*^
Income	−.128^*^	.168^**^	.153^*^	1	.086	.082	−.050	−.030	−.018	.002	.046	.052	.066
Cues	−.041	.298^**^	.088	−.004	1	.273^**^	.128	.166^*^	.146^*^	−.115	.122	.157^*^	.133^*^
Susceptibility	.126^*^	.168^**^	.009	−.002	.231^**^	1	.046	−.021	.072	−.010	.034	.028	.215^**^
Severity	−.013	.382^**^	.116	.129^*^	.157^**^	−.069	1	.462^**^	.031	−.102	.223^**^	.015	.005
Benefit	−.018	.234^**^	.037	.083	.204^**^	.109	.398^**^	1	−.269^**^	−.341^**^	.481^**^	.262^**^	−.050
Subjective barriers	−.053	−.110	−.066	−.050	.044	.005	−.135^*^	−.149^*^	1	.444^**^	−.304^**^	−.183^**^	.089
Objective barriers	.032	−.200^**^	−.136^*^	.050	−.107	.100	−.074	−.263^**^	.360^**^	1	−.362^**^	−.596^**^	−.052
Self−efficacy	.013	.156^*^	.070	.021	.024	.060	.206^**^	.504^**^	−.248^**^	−.372^**^	1	.236^**^	.005
SRB	.010	.142^*^	.163^**^	−.205^**^	.122^*^	.014	−.017	.090	−.148^*^	−.473^**^	.210^**^	1	−.096
24HUNa	−.036	.216^**^	.023	.062	.039	−.011	.053	.099	.010	−.097	.088	−.005	1

Note: * P<0.05; ** P<0.01; Bottom left – for rural participants; Top right – for urban participants.

Abbreviations: SRB = salt-restriction-spoon using behavior; 24HUNa = 24-hour urinary sodium.

As can be seen from Table 4, SRB and 24HUNa of participants from different areas were significantly correlated with different variables. Therefore, path analysis was conducted for rural and urban participants respectively.

### Exploratory path models for participants’ SRB and 24HUNa

**Figure 1:**
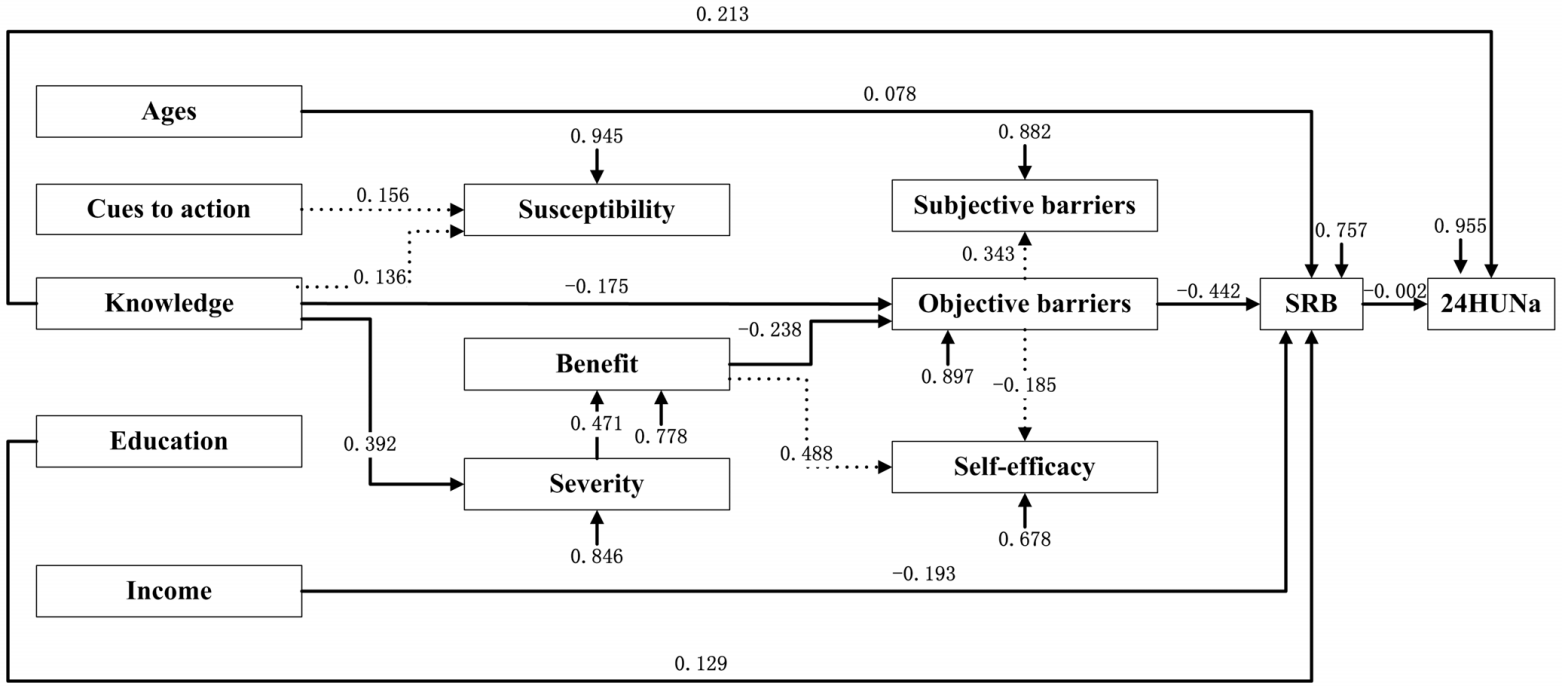
An exploratory path model for rural participants’ SRB and 24HUNa – standardized coefficients. Note: Goodness of Fit Index (GFI)  =  0.959; Root Mean Square Residual (RMR)  =  0.049; Chi-square  =  63.223, df  =  57, P  =  0.226; Bentler's Comparative Fit Index  =  0.991. SRB = salt-restriction-spoon using behavior; 24HUNa = 24-hour urinary sodium. The dotted line means the paths that will not finally arrive at SRB or 24HUNa.

**Figure 2:**
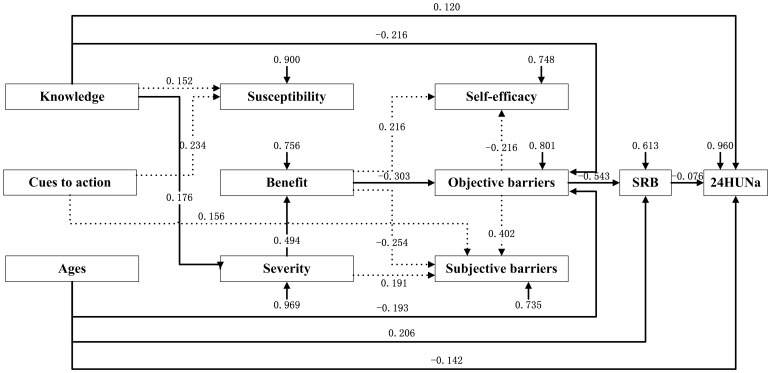
An exploratory path model for urban participants’ SRB and 24HUNa – standardized coefficients. Note: Goodness of Fit Index (GFI)  =  0.958; Root Mean Square Residual (RMR)  =  0.056; Chi-square  =  21.167, df  =  36, P  =  0.856; Bentler's Comparative Fit Index  =  0.999. SRB = salt-restriction-spoon using behavior; 24HUNa = 24-hour urinary sodium. The dotted line means the path that will not finally arrive at SRB or 24HUNa.

As depicted in Figure 1 and Figure 2, education, income, knowledge, cues to action and age were treated as a set of exogenous variables that were assumed to influence participants’ cognition toward hypertension, such as susceptibility, benefit and severity, which would then influence participants’ self-judgment on their feelings and efficacy (efficacy, objective barriers and subjective barriers), and finally cause effect on SRB and 24HUNa.Since education and income were significantly related to rural but not urban participants’ SRB, these two variables were only included in the analysis model for rural participants. The main indexes of goodness of fit indicate that the two models fit the data well (See notes for Figure 1 and Figure 2).

Contrary to our expectations, cues to action, susceptibility, self-efficacy and subjective barriers were not important exploratory variables for both rural and urban participants’ SRB and 24HUNa. For both rural and urban participants, SRB was negatively related to objective barriers (β =  − 0.442 and β =  − 0.543, respectively). In addition, benefit, severity, knowledge, and age were determinants for urban participants’ SRB and 24HUNa, while benefit, severity, knowledge, age, income and education for rural participants’ SRB and 24HUNa (refer to Figure 1 and Figure 2 for details).

## Discussion

This survey found that 36.1% of rural participants and 68.3% of urban participants used a salt-restriction-spoon every day, often or sometimes, while another survey among patients with primary hypertension conducted in Dalian, another city in China, indicated only a salt-restriction-spoon using rate of less than 5% [29]. These data indicate that the salt-restriction-spoon promotion in Beijing has achieved initial success, especially in urban areas. However, another survey conducted in Shanghai [30] found that as many as 84.6% of the residents choose to use a salt-restriction-spoon, indicating that there is still great potential for improvement in Beijing, especially in rural areas.

Corresponding to the enhanced usage rate of salt-restriction-spoon, the salt intake of Beijing residents has been reduced slightly. According to Table 3, the average 24HUNa for all participants was 162.80 mmol, equivalent to a salt intake of 11.07 g assuming that the average urinary excretion of sodium corresponds to 86% of total sodium intake [31]. Previous study conducted in Beijing [13] indicated a higher average level of 24HUNa (194.58 mmol). The Chinese National Nutrition and Health Survey conducted in 2002 also shown a higher average level of salt intake (12 g per day). However, efforts should be further made to lower salt intake, since current level is still much higher than the recommended amount of 6 g per day. According to this study, the average 24HUNa was 211.19 mmol for rural participants and 109.22 mmol for urban participants. The Chinese National Nutrition and Health Survey conducted in 2002 also shown a worse situation of rural residents by providing the result that 83.8% of rural residents and 76.8% of urban residents consumed more than the recommended amount of salt [13]. Therefore, rural residents should be considered as key target population of salt-restriction-spoon promotion.

The choice of HBM as a predicting model was based on its success in predicting other health-related behaviors and the fact that salt-restriction belongs to health behavior domain. As predicted by the HBM, individuals at older age, with more knowledge and higher education level would have better SRB. Therefore, education on the usage of SRB should be conducted especially among those who are relatively young but at risk of hypertension, and those who have lower education levels.

Contrary to the model’s prediction, cues to action, susceptibility, subjective barriers and self-efficacy failed to explain SRB of both rural and urban residents. Neyses [21] observed no significant correlation between nutritionists’ suggestion on salt-restriction (the suggestion can be treated as a cue to action) and healthy individuals’ actual sodium intake. The statement by Evers [20] that hypertensive patients’ response to nutrition advice was less favorable than expected also indicated that the inherent resistance to any change of lifestyle may require new motivational approaches.

Consistent with the model’s prediction, severity and benefit were indirectly related to SRB through the intermediate variable objective barrier (the design defect and using method of salt-restriction-spoon), which was the most significant influencing factor for both rural and urban residents’ SRB, indicating that the feasibility and simplicity of a salt-restriction tool are important factors for behavioral change. Past quantitative research reviews and meta-analyses undertaken on using the HBM with adults have shown that benefits, barriers, susceptibility and severity were very often significant predictors of behavior, in particular, barriers were the most reliable predictor followed by susceptibility, benefits and finally severity [32]. Similarly, in this study barriers were found to be the most consistent component, so we can argue that our results are partially in line with previous studies. Studies have shown that health benefits do not outweigh the importance of sensory properties of foods, and consumers are not ready to make sacrifices in hedonic pleasures [33], [34], indicating the difficulty in changing one’s taste habit (considered as a kind of subjective barrier in this study). The fact that perceived objective barriers rather than subjective barriers was the most important factor in predicting salt-restriction behavior suggests that participants classify salt-restriction as belongs to health domain instead of food domain and see it as an alternative to medicine that may help to lower the risk of diseases. Since perceived objective barriers have strong negative effect on individuals’ salt-restriction behavior, it is necessary to improve current salt-restriction-spoon by adding its feasibility and simplicity.

Contrary to our expectation, 24HUNa was poorly explained by SRB. Similar to our finding, Ohta [19] found that there was no obvious reduction in urine sodium excretion in salt-conscious patients. The weak correlation or non-correlation between salt-restriction behavior and urine sodium excretion may be due to the fluctuation in 24HUNa, which largely depended on participants’ diet on and before the day of urine collection. It may be necessary in further studies to add actual salt intake, an intermediate variable between salt-restriction behavior and urine sodium excretion, into the current HBM used in this study so as to explore the inner links.

Many countries have taken actions on salt-reduction. Some countries, such as the UK, USA and Finland, focus on encouraging food companies to reduce salt in processed, canteen, and restaurant food, but other countries, where salt intake comes mainly from home cooking, focus on encourage citizens to eat less salt [35]. It is exactly right for the Chinese government to hand out salt-restriction-spoon among citizens, since more than 85% of them eat at home at each meal. Other researchers have supported this point by stating that 76% of Chinese people’s dietary sodium came from salt added in home cooking [14]. Despite the fact that many people in Beijing have received a salt-restriction-spoon, some of them still refuse to use it. Based on the results and discussions above, we think it necessary to improve the current spoon (such as lengthen its handle, enlarge its caliber, make it more customer friendly, etc.) and to carry out corresponding education on the right usage of the spoon, the severity of hypertension, and the benefit of salt reduction, especially among those who are relatively young but at risk of hypertension, those who have lower education levels, and those who live in the rural areas.

There are several recommendations for future researches. First, this was a cross-sectional study, in which path analysis was conducted to ascertain the direction of influence among variables, but further longitudinal studies are needed to explain them more clearly. Second, objective barrier has been tested by the HBM to be the most important determinant of SRB, so an improved salt-restriction-spoon should be developed, and further intervention studies could be carried out to test the intervention effect. Third, it’s a little unexplainable that some variables, such as cues to action, susceptibility, subjective barriers, and self-efficacy cannot predict SRB and 24HUNa, further studies are necessary to verify these results.

In summary, the SRB can be predicted by the HBM, especially by some components such as perceived subjective barriers, as well as some demographic features (knowledge of hypertension, age, education, income, living areas). Improvement of the current salt-restriction-spoon and education on the right usage of the spoon, the severity of hypertension, and the benefit of salt reduction among some residents are necessary. Since some components of HBM seem to be ineffective, there is an opening for developing better models for explaining SRB.

## Limitations

One limitation of the study is that about 36% of the participants were excluded from the analysis because of incomplete or no submission of urine. Most of the excluded ones were in the relatively young age groups, and going to working in the day was the main reasons for their incomplete submission of urine. In analyzing the determinants of salt-restriction-spoon using behavior, the demographic features with significant difference were included in the models, thus the results would not be influenced by the high non-response rate. The second limitation of the study is that the overwhelming majority of participants were women, which has no substantial impact on the results of HBM score and 24HUNa of participants in both the rural and the urban areas because no significant difference in the above-mentioned indicators existed between women and men. The third limitation of the study is about the representative of the subject population. This study's research field is one of the biggest cities in the world, and convenience sampling was used as the sampling method in this study, therefore, the subject population may not represent the whole population. However, because not all cities in China have implemented the policy of sending salt-restriction-spoons among residents, only cities that have implement this policy can be chosen as the research field of our study. Beijing is the city implementing this policy in the earliest time, so it was set as our research field. This is the first attempt to analyze the influencing factor of salt-restriction-spoon using behavior in China. In the future, similar researches conducted in a wider range of areas using random sampling method can be considered so as to obtain a more reliable conclusion.
